# Sexual dimorphism in mitochondrial dysfunction and diabetes mellitus: evidence from a population-based cohort study

**DOI:** 10.1186/s13098-023-01090-1

**Published:** 2023-06-01

**Authors:** Shanjie Wang, JunChen Guo, Xiaoxuan Liu, Wei Tian, Yiying Zhang, Ye Wang, Yige Liu, Mingyan E., Shaohong Fang

**Affiliations:** 1 Department of Cardiology, Second Affiliated Hospital of Harbin Medical University, Harbin, 150000 China; 2 The Key Laboratory of Myocardial Ischemia, Chinese Ministry of Education, Harbin, 150000 China; 3 Department of Epidemiology and Biostatistics, School of Public Health, Jiamusi University, 154000 Jiamusi, China; 4 Department of Epidemiology and Biostatistics, School of Public Health, Harbin Medical University, Harbin, China; 5 Department of Thoracic Radiotherapy, Harbin Medical University Cancer Hospital, Harbin, Nangang District China

**Keywords:** Gender/Sex, Mitochondria, Methylmalonic acid, Cobalamin, Cardiometabolism, Mortality

## Abstract

**Background:**

Pathophysiological mechanisms underlying sex-based differences in diabetes remain poorly understood. Mitochondrial metabolite methylmalonic acid (MMA) accumulation reflects mitochondrial dysfunction which is involved in sex-specific pathophysiological responses biologically. We aimed to investigate the sex-specific associations between mortality risk and MMA in adults with the presence or absence of type 2 diabetes.

**Methods:**

This cohort study included 24,164 adults (12,123 females and 12,041 males) from the NHANES study during 1999–2014. Both sexes were separately categorized as those with no diabetes, prediabetes, undiagnosed diabetes, and diagnosed diabetes. Circulating MMA level was measured at baseline by mass-spectrometric detection. Mortality status was ascertained from baseline until December 31, 2015.

**Results:**

During a median follow-up of 11.1 years, 3375 deaths were documented. Males had a particularly higher mortality than females in adults with diagnosed diabetes compared to differences in those with no diabetes, prediabetes and undiagnosed diabetes (sex differences in mortality rate per 1000 person-years across diabetic status: 0.62, 1.44, 5.78, and 9.77, p < 0.001). Notably, the sex-specific difference in associations between MMA and mortality was significant only in adults with diagnosed diabetes (p for interaction = 0.028), not in adults with no diabetes and prediabetes. Adjusted HRs (95%CIs) per doubling of MMA for all-cause mortality were 1.19 (1.04–1.37) in females with diagnosed diabetes versus 1.58 (1.36–1.86) in male counterparts. In addition, MMA levels had an insignificant or weak correlation with sex hormone profiles at baseline, regardless of diabetes status and sex.

**Conclusions:**

Sex difference in mortality risk was especially significant in diagnosed type 2 diabetes. Increasing equivalent exposure to mitochondrial metabolite MMA was associated with a greater excess risk of future mortality in males with diabetes than in females.

**Supplementary Information:**

The online version contains supplementary material available at 10.1186/s13098-023-01090-1.

## Introduction

Sex-based difference remains conspicuous in metabolic syndrome and diabetes. In response to overnutrition, males are more likely to have obesity, insulin resistance, and type 2 diabetes than females [1]. Addressing sex-specific differences has emerged as a priority issue in diabetes. Current guidelines underscore the significance of elucidating sexual dimorphism in preclinical and clinical studies [1–3]. Although sex hormones are most commonly used to interpret the sexual difference in metabolic disorders, accumulative results are controversial. For example, estrogen confers premenopausal females with favourable cardiometabolic characteristics compared to those observed in males. However, some clinical studies have found a positive association between estrogen levels and diabetes risk in females [4, 5]. Male mice with gonadectomy show improved metabolic profiles similar to female mice, while testosterone in male humans is associated with cardiometabolic benefits [4, 6]. Results concerning the benefits of sex hormone therapy in clinical trials were also inconsistent [5, 7, 8]. Overall, clinical translation of sex hormones in sex-specific management seems difficult among patients with diabetes. The identification of novel risk factors to explain and solve the sex differences in diabetic progression is still important.

Mitochondrial dysfunction is one of the most striking characteristics in the pathogenesis of diabetes and associated complications [9, 10]. Mitochondria are involved in mediating sex-specific pathophysiological responses to metabolic disorders [9, 11]. Compared to females, mitochondrial dysfunction in males is associated with increased susceptibility to obesity and insulin resistance [9]. However, clinical evidence remains scarce. Methylmalonic acid (MMA) is a mitochondrial-derived intermediate metabolite that is best known as a functional indicator for cobalamin deficiency [12]. The metabolism of MMA is highly dependent on mitochondrial enzymes methylmalonyl-CoA mutase and coenzyme cobalamin. MMA accumulation caused severe mitochondriopathy by directly inhibiting the mitochondrial respiratory chain, α-ketoglutarate dehydrogenase, and mitochondria quality control. Hence, in addition to reflecting cobalamin status, circulating MMA is also considered a surrogate marker of mitochondrial dysfunction [13–17]. Decreasing MMA levels have been shown to alleviate mitochondria-rich organs kidney and brain in children and animal models of inherited methylmalonic aciduria [18]. Thus, MMA may be a potentially modifiable marker reflecting mitochondrial dysfunction to explain the worsened prognosis in males with diabetes. In this study, we investigated the sex difference in mortality rate in the presence or absence of diabetes, as well as the sex-specific associations between serum levels of MMA and mortality risk.

## Methods

### Study design and population

National Health and Nutrition Examination Survey (NHANES) comprises complex-survey samples of a nationally representative population of noninstitutionalized U.S. civilians. NHANES study was a large prospective cohort in which participants were linked to mortality outcomes [18]. The data in NHANES were collected in each 2-year cycle since 1999. A total of 25,131 participants 18 of age or older who were eligible for MMA determination were included, because serum MMA was only measured in participants recruited in 5 study cycles (1999–2000, 2001–2002, 2003–2004, 2011–2012, and 2013–2014). After excluding participants who were pregnant (n = 886) or had missing covariates of diabetes status (n = 6), possible type 1 diabetes (aged less than 20 years with only insulin for antidiabetic treatment, n = 45)[19], or missing data for mortality status (n = 30), 24,164 adults were included for analysis.

We categorized diabetes status into four groups: previously diagnosed type 2 diabetes (i.e., diagnosis by doctors and/or the use of antidiabetic agents, n = 2535), undiagnosed diabetes (i.e., no previous diagnosis, no use of antidiabetic medication, and plasma HbA1c levels of ≥ 6.5% and/or fasting plasma glucose (FPG) of ≥ 126 mg/dL, n = 776), prediabetes (i.e., no previous diagnosis, no use of antidiabetic medication, and plasma HbA1c levels in the ranges of 5.7–6.5% and/or FPG in the ranges of 100–126 mg/dL, n = 5406), no diabetes (i.e., no previous diagnosis, no use of antidiabetic medication, HbA1c < 5.7% and FPG < 100 mg/dL, n = 15,447).

### Measurement of serum methylmalonic acid

As mentioned in our previous reports [18, 19], MMA levels were measured in venous blood using gas chromatography-mass spectrophotometry (GC/MS) in participants during 1999–2004 and measured by liquid chromatography-mass spectrophotometry (LC-MS/MS) in participants during 2011–2014. High-performance GC/MS (Model 6890 GC system and Model 5973 mass selective detector, Hewlett-Packard, San Fernando, CA) was applied for MMA determination in NHANES 1999–2004. In brief, 275 µL specimens with internal standard solution supplemented by isotope-labelled methyl-d^3^-malonic acid (d^3^MMA) were extracted and subsequently derivatized with cyclohexanol to produce a dicyclohexyl ester. The chromatography was performed on a DB-5MS capillary GC column (0.25 mm x 30 m, 0.25-µm, J&W Scientific, Folsom, CA) within 15 min. The effluent part from the GC column was monitored with a mass-selective detector by the selected ion monitoring process. MMA levels were quantitated using the peak area ratios of MMA and the internal standard, isotope-labelled d^3^MMA. LC-MS/MS method was established for MMA detection in NHANES 2011–2014 using Thermo-Electron HPLC System and Thermo-Electron TSQ triple quadrupole mass spectrometer (Thermo Scientific, West Palm Beach, FL.). Compared to GC/MS procedures, LC-MS/MS method required less sample volume (75ul) and a shorter run time (6 min). MMA is extracted from 75 µL of sample along with an added internal standard (d3-MMA) via liquid-liquid extraction method with tert-butylmethylether. Then, the extracted organic acid is derivatized with butanol to form a dibutylester. The Hypersil Gold C18 column (2.1 mm × 50 mm, 1.9 μm particle size, Thermo Fisher Scientific) was selected because it allowed an excellent separation between MMA and succinic acid with relatively short chromatography run time and narrow analyte peaks. The butanol is evaporated under vacuum and the derivatized sample is reconstituted in acetonitrile/water (v/v 50/50). MMA was chromatographically separated from succinic acid using isocratic mobile phase (0.1% acetic acid: methanol 40:60 (v/v), 0.4 mL/min, 35 °C) within 6 min (retention time 3.47 min for SA and 4.25 min for MMA). Multiple reaction monitoring (MRM) was performed in positive electrospray ionization mode, with two transitions each for MMA (m/z 231 → 119 and 175.1) and for d3-MMA (m/z 234.1 → 122.1 and 178.1). Both methods showed excellent correlation and agreement [20]. MMA in the range of 50–2000 nmol/L has a favourable linear pattern with detectable signal intensity. Samples with MMA levels of more than 400 nmol/L were repeatedly analyzed to eliminate the detection error. The total coefficient of variation ranged from 4 to 10%, and the recovery rate was 94.0–96.0% in each study cycle. Detailed protocols and procedures have been described elsewhere [19].

### Study outcomes

Participants in NHANES were linked to the National Death Index to identify vital status. The publicly used mortality data were recorded from baseline until death or December 31, 2015. The detailed protocols of the linkage and analysis guidelines for mortality data have been published by the National Center for Health Statistics [18]. Mortality attributed to heart disease was identified according to the International Classification of Diseases, 10th Revision (ICD-10), including I00-I09, I11, I13, and I20-I51.

### Other variables

General characteristics at baseline, including age, sex, race/ethnicity (non-Hispanic White, non-Hispanic Black, Hispanic, or others), and smoking status (never, former, current) were reported by participants using standardized questionnaires [19]. Adequate physical activity was defined as at least 150–300 min/week of moderate-intensity, 75–150/week min of vigorous physical activity, or equivalent combination of moderate and vigorous physical activity. Body mass index (BMI) was calculated as weight (in kilograms) divided by height (in meters squared). Systolic/diastolic blood pressure was calculated as the mean of three eligible values. Hypertension was defined as the use of antihypertensive therapy or average blood pressure ≥ 140/90 mmHg. Chronic kidney disease (CKD) was defined as an estimated glomerular filtration rate < 60 mL/min/1.73 m^2^ using the Chronic Kidney Disease Epidemiology Collaboration formula. Cardiovascular disease (CVD) included self-reported coronary heart disease, heart failure, or stroke. Cancer was defined as self-reported cancer or a malignant tumour diagnosed by a physician. Data concerning the duration of diabetes, antidiabetic medications, and peripheral complications in persons with diabetes (e.g., foot ulcer/sore, meroparesthesia, and retinopathy) were extracted from diabetes questionnaires. Menopause was defined as amenorrhoea longer than 12 months attributed to menopause/hysterectomy.

All assessments of biospecimens were performed in the central laboratory. Total cholesterol (TC), high-density lipoprotein cholesterol (HDL-C), HbA1c, plasma glucose, and serum creatinine levels were measured according to validated laboratory methods during each study cycle. C-peptide (n = 6,726), insulin (n = 11,606), serum total testosterone (TT, n = 11,232), estradiol (n = 6,309), and sex hormone-binding globulin (SHBG, n = 5,797) levels were only measured in a subset. Free testosterone levels were estimated using the empirical free testosterone formula, as previously reported [21]. Serum cobalamin (vitamin B12) levels were measured using the commercial radioassay kit (Bio-Rad Laboratories, 1993) from 1999 to 2004 and using the automated electrochemiluminescence immunoassay (Roche, Elecsys 170) from 2011 to 2014. Both assays were calibrated to correct for changes across years according to NHANES recommendations [19].

### Statistical analysis

According to the analytical guidelines of the NHANES study, clustering, strata, and sample weights were recorded to account for the complex sampling design and nationally representative estimates, unless otherwise noted [18]. Variables are expressed as weighted means (standard error, SE) and percentages. At the baseline, sex-specific characteristics were described by diabetes status. The generalized linear model was used to estimate the means of blood metabolic indicators after adjustment for age, sex, race/ethnicity, BMI, smoking, hypertension, and eGFR. The correlation coefficients between serum sex steroid hormones (total testosterone, free testosterone, estradiol, and SHBG) and MMA were estimated using the Spearman method. The correlation analysis between MMA and sex hormone profiles was repeated after adjustment for menopause status in females.

Sex-specific mortality rates across diabetes groups were estimated based on the Poisson distribution method and presented as events per 1000 person-years of follow-up. Cox proportional hazard regression analysis was used to estimate hazard ratios (HRs) and 95% confidence intervals (CIs) for the mortality risk per doubling of serum MMA (each unit increase of log2 transformed values of MMA), which were stratified by diabetes status and sex. The proportional hazards assumption was tested and fulfilled. In the multivariate analyses, model 1 was adjusted for age, and model 2 was further adjusted for race/ethnicity, smoking status, physical activity, BMI, hypertension, cancer, CVD, the ratio of TC/HDL-C, eGFR, plasma HbA1c, and cobalamin. The significance of the interaction between MMA levels and sex for mortality risk was assessed via the weighted Wald test.

Several sensitivity analyses were conducted. First, to examine the dose-response relationship between serum MMA concentrations and mortality risk by diabetes status and sex, a restricted cubic spline with four knots (5th, 35th, 65th, and 95th ) was used based on the unweighted Cox regression model with multivariate adjustment as mentioned above. Second, the analysis for adults with diagnosed diabetes was additionally adjusted for diabetes-related variables, including the duration of diabetes, diabetic complications (including diabetic limb ulcer/sore, retinopathy, and peripheral neuropathy), UACR (mg/g), metformin use, ACEI/ARB, and blood-lipid lowering drugs. We further reanalyzed participants with eligible sex hormone profiles to assess whether sex hormones modified the relationship between MMA and mortality risk in males and females, and menopausal status was considered for female participants. All tests with a 2-sided p value less than 0.05 were considered statistically significant using Stata (version 12).

## Results

### Characteristics of participants according to diabetic status and sex

A total of 24,164 individuals (12,123 females and 12,041 males) were included in this study, with mean ages of 47.4 and 45.3 years for females and males, respectively. Overall, 19.8% were characterized as prediabetes, 2.4% had incident diabetes and 7.8% were previously diagnosed with diabetes. Baseline characteristics by diabetes status and sex are presented in Table 1. In both males and females, participants with prediabetes or diabetes were older, less likely to have smoking or adequate physical activity, more likely to have chronic diseases, and had higher levels of BMI, waist circumference, and eGFR compared to adults with no diabetes. Furthermore, current smoking, diabetic complications and UACR were more common in males with diagnosed diabetes, while obesity, hypertension, and CKD were more prevalent in females with diabetes than males.

**Table 1:** Baseline characteristics of participants by diabetes status and sex

	Females				Males			
**Variables**	No diabetes (n = 7,956)	Prediabetes (n = 2,578)	Undiagnosed diabetes (n = 339)	Diagnosed diabetes (n = 1,250)	No diabetes (n = 7,491)	Prediabetes (n = 2,828)	Undiagnosed diabetes (n = 437)	Diagnosed diabetes (n = 1,285)
Age (year)	43.1 ± 0.32	57.0 ± 0.32	57.9 ± 1.02	60.4 ± 0.46	40.9 ± 0.29	52.8 ± 0.40	56.4 ± 0.97	59.6 ± 0.49
Race/ethnicity (%)								
Non-Hispanic White	71.82	65.99	50.42	59.61	72.24	62.97	56.50	64.74
Non-Hispanic Black	9.51	14.99	20.83	17.21	8.318	15.34	11.61	12.80
Hispanic-Mexican	7.16	6.33	8.92	8.81	8.40	8.92	11.14	8.14
Other	11.52	12.68	19.84	14.36	11.04	12.77	20.74	14.32
Smoking status (%)								
Never	59.54	58.91	61.59	56.44	48.26	38.40	40.76	38.55
Former	19.09	23.36	24.53	27.16	25.31	35.05	38.10	42.28
Current	21.37	17.73	13.88	16.40	26.43	26.55	21.13	19.17
Adequate physical activity (%)	50.09	35.38	24.77	29.97	60.23	43.75	36.13	37.05
BMI (kg/m2)	27.2 ± 0.11	31.1 ± 0.21	34.0 ± 0.62	33.3 ± 0.28	27.2 ± 0.07	29.6 ± 0.18	32.7 ± 0.64	31.9 ± 0.36
Normal weight	43.46	21.54	12.86	13.52	35.25	20.28	8.56	13.93
Overweight	29.02	28.88	24.98	24.15	40.48	40.53	31.63	30.87
Obesity	27.52	49.58	62.16	62.33	24.27	39.18	59.81	55.20
Hypertension (%)	26.94	56.00	65.89	75.07	28.32	49.19	55.08	68.61
Systolic BP, mmHg	118.1 ± 0.29	129.7 ± 0.56	133.6 ± 1.13	133.2 ± 0.87	121.5 ± 0.30	126.9 ± 0.48	132.5 ± 1.80	130.0 ± 0.63
Diastolic BP, mmHg	70.1 ± 0.23	71.1 ± 0.38	70.5 ± 1.07	67.0 ± 0.63	72.4 ± 0.23	73.1 ± 0.41	74.8 ± 1.14	71.0 ± 0.57
CVD (%)	4.97	11.30	14.40	27.94	5.7140	16.19	15.61	26.99
Cancer (%)	8.091	13.53	12.6	16.12	5.862	12.42	4.932	16.11
eGFR (mL/min per 1.73 m²)	101.1 ± 0.45	87.9 ± 0.55	90.5 ± 1.81	82.1 ± 1.00	99.7 ± 0.40	90.0 ± 0.56	91.6 ± 1.34	83.9 ± 0.85
CKD (%)	4.56	11.73	14.23	21.35	3.07	9.92	6.89	18.67

Data are presented as weighted proportions (%) or means ± standard error (SE). BMI, body mass index; BP, blood pressure; CKD, chronic kidney disease; CVD, cardiovascular disease; eGFR, estimated glomerular filtration rate, UACR, urinary albumin-to-creatinine ratio

The means of metabolism-related circulating biomarkers according to diabetic status and sex after correcting for age, race/ethnicity, BMI, smoking, hypertension, and eGFR levels are presented in Table 2. Compared to participants with no diabetes or prediabetes, those with undiagnosed or diagnosed diabetes had a higher burden of inflammation and cardiometabolic disorders in both sexes. For instance, an increase in plasma CRP, fasting glucose, insulin, C-peptide, TG, and TC/HDL-C ratio was observed (each p-trend < 0.001). Among participants with diabetes, males had higher levels of blood glucose, HOMA-IR and TC, while females had higher levels of CRP, TC and LDL-C.

**Table 2:** Multivariable-adjusted means of glucolipid metabolic markers by diabetes status and sex

	Females				Males			
	No diabetes	Prediabetes	Undiagnosed diabetes	Diagnosed diabetes	No diabetes	Prediabetes	Undiagnosed diabetes	Diagnosed diabetes
CRP, mg/dL	0.5 ± 0.01	0.6 ± 0.03	0.7 ± 0.07	0.7 ± 0.04	0.3 ± 0.01	0.4 ± 0.02	0.6 ± 0.06	0.5 ± 0.04
HbA1c, %	5.2 ± 0.01	5.8 ± 0.02	7.3 ± 0.04	7.4 ± 0.02	5.3 ± 0.01	5.7 ± 0.01	7.4 ± 0.04	7.5 ± 0.02
HbA1c, mmol/mol	33.7 ± 0.10	39.7 ± 0.17	56.4 ± 0.45	57.6 ± 0.24	33.9 ± 0.1	39.1 ± 0.16	56.9 ± 0.39	58.4 ± 0.24
Insulin, pmol/L	65.6 ± 1.34	85.8 ± 2.11	111.8 ± 5.70	124.6 ± 3.29	71.4 ± 1.97	84.0 ± 2.67	108.3 ± 7.14	126.8 ± 4.56
C-peptide (nmol/L)	0.8 ± 0.01	1.0 ± 0.02	1.1 ± 0.04	1.0 ± 0.02	0.8 ± 0.01	1.0 ± 0.02	1.1 ± 0.04	1.1 ± 0.03
HOMA-IR index	2.0 ± 0.05	3.1 ± 0.07	6.0 ± 0.20	5.5 ± 0.12	2.2 ± 0.10	2.9 ± 0.13	6.0 ± 0.35	6.3 ± 0.24
Glucose, mmol/L	4.9 ± 0.02	5.5 ± 0.03	8.2 ± 0.09	8.4 ± 0.05	5.0 ± 0.02	5.6 ± 0.03	8.4 ± 0.08	8.7 ± 0.05
HDL-C, mmol/L	1.5 ± 0.01	1.4 ± 0.01	1.3 ± 0.02	1.3 ± 0.01	1.3 ± 0.01	1.2 ± 0.01	1.2 ± 0.02	1.2 ± 0.01
LDL-C, mmol/L	3.0 ± 0.02	3.1 ± 0.03	3.0 ± 0.07	2.7 ± 0.04	3.0 ± 0.02	3.1 ± 0.02	3.0 ± 0.07	2.6 ± 0.04
TG, mmol/L	1.4 ± 0.01	1.6 ± 0.03	2.0 ± 0.07	2.1 ± 0.04	1.7 ± 0.02	1.8 ± 0.03	2.4 ± 0.08	2.2 ± 0.05
TC, mmol/L	5.1 ± 0.01	5.2 ± 0.02	5.1 ± 0.06	4.9 ± 0.03	5.0 ± 0.01	5.1 ± 0.02	5.1 ± 0.05	4.6 ± 0.03

CRP, C-reactive protein; HbA1c, glycosylated hemoglobin; HOMA-IR, homeostasis model assessment for insulin resistance; HDL-C, high-density lipoprotein cholesterol; LDL-C, low-density lipoprotein cholesterol; TG, triglyceride; TC, Total cholesterol. All estimates were calculated after adjustment for age, race/ethnicity, BMI, smoking, physical activity, hypertension, and eGFR. P trend for each biomarker across diabetes status by sex was less than 0.001 estimated by multiple linear regression

**Fig. 1:**
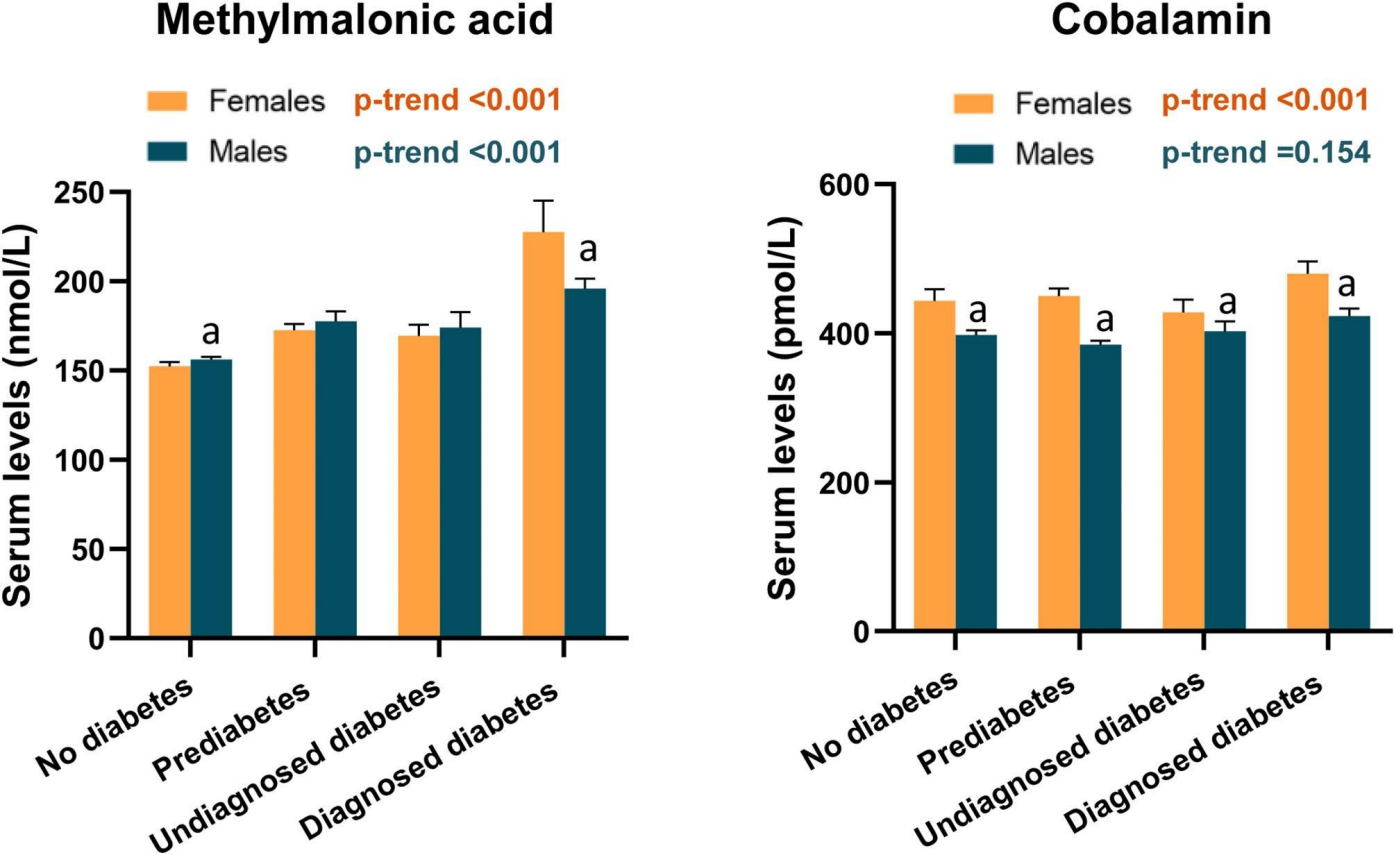
Circulating levels of MMA and cobalamin by diabetes status and sex The estimates of serum cobalamin (vitamin B12) and MMA were weighted with the complex sampling design and represented as mean ± SE. p values were estimated by Student’s t-test. p-trend across diabetic status was estimated by a weighted linear regression model. ^a^ p < 0.05 for men versus women

Notably, sex-related differences in MMA metabolism were significant among participants with diabetes (Fig. 1). Circulating MMA concentrations were significantly elevated across diabetic status for both male and female sexes (p < 0.001); however, unexpectedly, females with diabetes showed higher MMA levels than males (weighted means: 227.5 versus 195.9 nmol/L, p < 0001). Serum cobalamin levels did not change significantly in males with no diabetes, prediabetes, or diabetes (p for trend = 0.154). In contrast, an increase in serum cobalamin levels was noted in females with no diabetes to those with diagnosed diabetes (p-trend < 0.001). Cobalamin levels were always lower in males than in females, regardless of diabetic status.

### Sexual differences in associations between MMA and mortality were significant among participants with diagnosed diabetes

Over a median follow-up of 11.1 years, 3375 deaths were documented, including 766 and 700 cardiovascular disease- and cancer-related deaths, respectively. The weighted mortality increased by five times from males and females who did not have diabetes to those who were diagnosed with diabetes (Table 3). Notably, compared to sex difference in mortality in participants with no diabetes, prediabetes or undiagnosed diabetes, the mortality rate especially increased in males with diagnosed diabetes than in females. Men-to-women differences in mortality rates from no diabetes, prediabetes, and new-diagnosed diabetes to previously diagnosed diabetes groups were 0.62, 1.44, 5.78, and 9.77, respectively.

**Table 3:** Sex-specific all-cause mortality risk associated with serum MMA according to diabetes status

		Deaths/ person-years ^a^	Mortality rate ^b^ (95%CI)	Rate Difference	Age-Adjusted HR (95%CI)	p value	Multivariable-adjusted HR (95%CI)	p value	p-interaction
No diabetes									
	Females	726/73,992	7.32 (6.70–8.02)	0.62 (0.35–0.90)	1.38 (1.24–1.54)	< 0.001	1.29 (1.13–1.47)	< 0.001	0.782
	Males	862/69,463	7.94 (7.28–8.68)		1.39 (1.27–1.53)	< 0.001	1.34 (1.19–1.52)	< 0.001	
Prediabetes									
	Females	382/18,222	17.57 (15.56–19.90)	1.44 (0.65–2.21)	1.51 (1.28–1.79)	< 0.001	1.49 (1.26–1.78)	< 0.001	0.108
	Males	528/20,060	19.01 (17.01–21.28)		1.39 (1.21–1.61)	< 0.001	1.30 (1.08–1.58)	0.007	
Undiagnosed diabetes									
	Females	64/2,518	19.66 (14.52–27.02)	5.78 (3.61–7.95)	2.33 (1.69–3.21)	< 0.001	1.50 (1.01–2.23)	0.045	0.186
	Males	102/3,148	25.44 (19.77–33.08)		1.84 (1.45–2.34)	< 0.001	1.66 (1.12–2.51)	0.015	
Diagnosed diabetes									
	Females	309/8,720	32.38 (28.11–37.43)	9.77 (8.33–11.21)	1.26 (1.07–1.48)	0.007	1.19 (1.04–1.37)	0.014	0.028
	Males	402/8,484	42.15 (37.14–47.97)		1.61 (1.42–1.83)	< 0.001	1.58 (1.36–1.86)	< 0.001	

^a^ unweighted, ^b^ weighted death rate per 1000 person-year. HR, hazard ratio; CI, confidence interval. Multivariable-adjusted model was adjusted for age (continuous), race/ethnicity (non-Hispanic white, non-Hispanic black, Hispanic-Mexican and other), smoking status (never, ever, and current), Adequate physical activity(no/yes), BMI (normal, overweight, and obesity), hypertension (no/yes), cancer (no/yes), CVD (no/yes), TC/HDL-C ratio (continuous), eGFR (continuous), HbA1c (continuous), and serum cobalamin (continuous). Mortality rate was calculated according to the Poisson distribution. HR (95%CI) and p-value were calculated by weighted Cox proportional hazards regression. The interaction between MMA and sex was assessed by survey-weighted Wald test

As expected, the sex-specific associations between serum cobalamin and mortality were not noted in adults with or without diabetes. Given that MMA metabolism was influenced by ageing [22], we assessed the sex-specific association between MMA and mortality risk in age-adjusted Cox regression models. Baseline MMA was significantly associated with a higher risk of all-cause mortality in both sexes, from those who were not diagnosed with diabetes to those diagnosed with diabetes (each p ≤ 0.007, Table 3). After full adjustment for age, race/ethnicity, smoking status, physical activity, BMI, hypertension, TC/HDL-C ratio, CVD, cancer, eGFR, HbA1c and serum cobalamin, HRs (95% CIs) per doubling of MMA in females with no diabetes, prediabetes, undiagnosed diabetes and diagnosed diabetes were 1.29 (1.13–1.47), 1.49 (1.26–1.78), 1.50 (1.01–2.23) and 1.19 (1.04–1.37), respectively (Table 3). Multivariable-adjusted HRs (95% CIs) per doubling of MMA for all-cause mortality in males were 1.34 (1.19–1.52), 1.30 (1.08–1.58), 1.66 (1.12–2.51), and 1.58 (1.36–1.86), respectively. Sex-specific differences in the association between MMA levels and mortality risk were insignificant among participants with normoglycemia and prediabetes (each p for interaction ≥ 0.108), while the interaction between mitochondrial MMA and sex on mortality was significant in adults with diagnosed diabetes (p for interaction = 0.028). With each unit increase of log2-transformed MMA, mortality risk increased by 19% in females and 58% in males who had previously been diagnosed with diabetes. Similar associations were observed between serum MMA and mortality due to heart disease (**Table **S1).

**Fig. 2:**
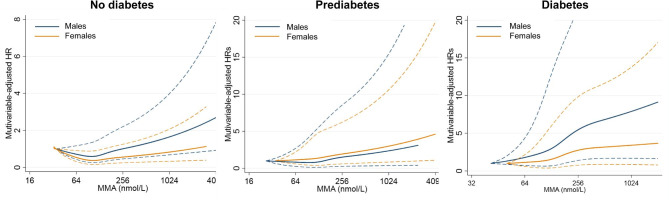
The sex-specific association of all-cause mortality with MMA levels across diabetes status The restricted cubic spline curve showed the adjusted hazard ratios in adults with **(A)** no diabetes, **(B)** prediabetes, and **(C)** diabetes for all-cause mortality based on the Cox regression model (unweighted). The model was fully adjusted for age, race/ethnicity, smoking, physical activity, BMI, hypertension, cancer, CVD, TC/HDL-C ratio, eGFR, HbA1c, and serum cobalamin. The solid line represented point estimates, and the dashed lines represented 95% CIs.

According to the spline fitting, linear associations between MMA levels and all-cause mortality were observed in the presence or absence of diabetes. However, the slope was particularly steeper among males with diabetes than among females (Fig. 2). When adjusted additionally for diabetes-related characteristics in the weighted Cox regression model, the increased mortality risk per doubling of MMA remained higher in males with diabetes than in females (**Table **S2 and S3), with HRs of 1.41 versus 1.08 for all-cause mortality (p-interaction = 0.036) and 1.72 versus 1.12 for heart disease mortality (p-interaction = 0.031).

### Sex steroid hormones may not explain the sex-specific association between mitochondrial MMA and mortality risk in diabetes

Sex-specific differences in the pathophysiology of diabetes are traditionally attributed to sex steroid hormones. Of the 24,164 participants, 11,232 received blood tests for at least one serum steroid hormone. Serum concentrations of total and free testosterone and SHBG significantly decreased from no diabetes and prediabetes to diabetes group in both males and females (**Figure **S1). However, decreased levels of estradiol across diabetic statuses were observed only observed in females and not males. Age-standardized levels of estradiol in participants with normoglycemia versus diagnosed diabetes were 230.1 pmol/L versus 155.4 pmol/L for females (p < 0.001) and 101.1 pmol/L versus 107.3 pmol/L for males (p = 0.094). The correlation coefficients between sex hormone profiles and MMA levels were insignificant or weak (**Table **S4). The Spearman’s rho was largely similar between females with menstrual cycles and those with menopause (**Table **S5). When additionally adjusted for total testosterone, free testosterone, estradiol, SHBG, or both free testosterone and estradiol in multivariable-adjusted Cox regression analysis, the association between the accumulation of mitochondria-derived MMA and mortality risk remained unchanged for females or males (**Table **S6).

## Discussion

In this large prospective cohort study, sexual differences in mortality risk were especially significant among participants with diagnosed diabetes compared to those with no diabetes or prediabetes. This finding may be explained by the interactive effect between MMA and sex on future mortality among adults with diagnosed diabetes. Nonetheless, MMA-associated mortality remained changed after adjustment for sex hormones in males or females. To our knowledge, this is the first study to provide population-based evidence supporting sexual dimorphism in mitochondrial-derived metabolite and diabetic progression.

Consistent with previous reports [23], we observed that males had a significantly higher risk of all-cause and cardiac mortality than females with or without diabetes. Males with type 2 diabetes were associated with a higher risk of micro- and macro-vascular complications than females with diabetes [24–26]. These findings support the hypothesis of cardioprotective effects associated with the female sex during diabetes progression [26]. Although the absolute incidence of adverse outcomes was higher in males with diabetes, some studies observed that diabetes was associated with an increased relative risk of cardiovascular events in females compared to that in males [27–29]. This finding may be attributed to worse cardiometabolic profiles and cardiovascular disease burden, less use of prescription medications, and poor medication adherence [26, 29]. Our study also observed higher BMI levels in females than males with diabetes. We did not focus on sex differences in the relative risk of mortality increased by diabetes but sought to investigate the mechanism concerning the higher mortality rate in males with diabetes from the perspective of mitochondrial metabolite.

Although MMA was a traditional indicator of cobalamin deficiency, our previous studies only observed a moderate correlation between circulating MMA and cobalamin (r = 0.3). We recently found that serum MMA, but not serum cobalamin or dietary cobalamin intake, was significantly associated with mortality risk in patients with diabetes [19]. Moreover, half of diabetic patients with MMA accumulation did not have cobalamin deficiency [19]. Functional cobalamin deficiency was used to describe a condition in which both MMA and B12 are elevated. Although the specific mechanism is unclear, more attention should be paid to mitochondrial MMA metabolism itself for MMA increase in addition to cobalamin deficiency. Numerous studies demonstrated that MMA was a surrogate biomarker of mitochondrial dysfunction in humans and animals [13–16, 30–32], which was more stable than mitochondrial oxidative products as a biomarker for mitochondria status [18]. Mitochondria have been considered the key regulatory centres located at the crossroads of complex cellular processes with myriad biochemical roles [33]. Our previous reports demonstrated the critical role of mitochondrial impairment in the pathology of diabetic cardiomyopathy [10, 34]. Our results supported the sexual dimorphism in mitochondrial pathophysiology which was well-established in animals with diabetes; however, clinical evidence remains scarce [1]. Although cobalamin deficiency, ageing, and renal dysfunction were the risk factors of MMA accumulation, this study demonstrated a robust association between MMA and cardiovascular mortality independent of age, serum cobalamin and eGFR levels [18, 19]. Ageing, cobalamin deficiency and renal dysfunction cannot explain this link. MMA accumulation may directly cause mitochondrial dysfunction in the cardiovascular system and adverse outcomes. Other reports from the NHANES study reported nonsignificant sex differences in multivariate-adjusted serum MMA concentration [35]. In our study, females with diabetes seemed to have moderately higher MMA levels than males with diabetes. This seemingly contradictory difference may be explained by the fact that females are more likely to be elder or have obesity hypertension, cardiovascular disease, and chronic kidney disease than males with diabetes. Notably, mortality risks increased by 58% in males with diabetes but only 19% in females with diabetes per each one-unit increase in log2-transformed MMA after adjustment for age eGFR and serum cobalamin.

Sexual dimorphism in mitochondrial biology and metabolic disorders has been identified in animals [9, 36, 37]. Males with mitochondrial dysfunction are specifically susceptible to obesity-related insulin resistance and metabolic dysregulation [9, 38]. Under conditions involving stress, female animals present more functional mitochondria than males [36]. A multi-omics analysis observed a downregulated metabolism involving the conversion of MMA-CoA to succinyl-CoA in obese subjects compared to that in lean subjects, suggesting an association between adiposity-related mitochondrial dysfunction and MMA accumulation [9, 39, 40]. Frode and colleagues systematically validated the sex difference in the oxidative function of isolated mitochondria in mice, which suggested a hub role of mitochondria in sexual differences and cardiometabolic disorders [9]. Our findings further provided population-based evidence showing that there was a sexual dimorphism between mitochondrial dysfunction, which was assessed by MMA accumulation, and the long-term mortality risk among adults with diabetes. For the general population with MMA accumulation with cobalamin deficiency, cobalamin supplementation remains preferred. Liver transplantation, Mmut gene transfection (to promote MMA metabolism), and a protein-restricted diet (to reduce MMA sources) have been shown to alleviate damage to mitochondria-rich organs in children and animal models of inherited methylmalonic aciduria [18]. Mitochondria may be a promising target of sex-specific biology and management in diabetes.

Gonadal hormones, the most common reason to explain the sexual discrepancy, may not completely account for the sexual dimorphism in mitochondrial traits [1]. Estrogens are generally considered to facilitate mitochondrial homeostasis, whereas the conclusions on testosterone in mitochondrial biology remain controversial [9, 38]. Pitteloud et al. found that low serum testosterone levels are associated with impaired mitochondrial function and insulin resistance in the skeletal muscle of 60 males [41]. Notably, reverse causality should be noted, because diabetes and mitochondrial dysfunction accelerate biological ageing that also mediates a decrease in testosterone levels [42]. Additionally, an increased risk of metabolic disorder and cardiovascular death has been observed in adults with sex steroid treatment [5, 8]. Thus, clinical translation of targeting the sex hormone axis for sex-based management of diabetes seemed unlikely. We did not observe the confounding effect of steroid hormones on the relationship between mitochondrial MMA and mortality in males or females. sex chromosomes regulating mitochondrial function independently of sex hormones, as well as the role of sex-related external factors, such as lifestyle, environmental exposure and dietary pattern, may not be ignored according to previous reports [38, 43]. The correlation between sex hormone profiles and MMA was also weak or insignificant. Overall, the metabolism of mitochondrial MMA as a target may contribute to new insights into the sex-specific management of diabetes.

### Strengths and limitations

The strengths of this study include data from a nationally representative sample that was collected via well-designed and validated protocols, which facilitate the generalization and reproducibility of our findings. Several limitations should be considered. First, both MMA and cobalamin levels were determined between 1999 and 2004 and 2011–2014 using two different assays. This may be of limited concern because previous studies demonstrated the comparability of those methods. Second, Although the link between MMA and mitochondrial dysfunction and structural abnormalities has been confirmed by pathological studies in humans, a single biomarker is insufficient to comprehensively assess mitochondrial status. Mitochondria were considered the key regulatory pivot located at the crossroads of complex cellular processes with myriad biochemical roles. A panel comprising indicators reflective of multidimensional mitochondrial performances (e.g., muscle biopsy, circulating mtDNA, and proteins and metabolites associated with mitochondrial metabolism) is a better approach in further studies. Third, sex hormone measurements in NHANES were limited to a subset of the population. Hence, we could not comprehensively estimate the influence of androgens and estrogens on the sex-based association between mitochondria and mortality risk in diabetes. However, the relationship between MMA and mortality remained unchanged in males and females after further adjustment for sex hormones. Fourth, transgenders account for approximately 0.6% of the U.S. population [43]. Potential transgender participants in NHANES may not strictly distinguish between gender and sex in the demographic questionnaire, but this tends to bias the sex-specific difference toward the null.

## Conclusions

 Sexual differences in mortality risk were especially significant among adults with diagnosed diabetes compared to participants with no diabetes or prediabetes. Moreover, we found that increased mortality risk associated with mitochondrial metabolite MMA was significantly higher in males with diabetes than in females, indicating that mitochondrial MMA was a modifiable marker in known sexual dimorphism in diabetes. Our findings recommended further investigation of the biological mechanisms of mitochondria-derived metabolite MMA in sex dimorphism.

## Electronic supplementary material

Below is the link to the electronic supplementary material.

Figure S1. Standardized levels of Serum Sex Hormones and SHBG by sex and diabetes status. Table S1. HRs for heart-specific mortality per each unit increase in log2-transformed MMA among adults with diabetes. Table S2. Diabetes-related variables at basline by sex. Table S3. The HRs for all-cause and heart-specific mortality per each unit increase in log2-transformed MMA after additional adjustment for diabetes-related variables. Table S4. Correlations of methylmalonic acid with serum sex hormones and SHBG by sex and diabetes status. Table S5. Spearman's rho between methylmalonic acid and sex hormones in females by menopause status. Table S6. Sex-specific HRs for the relationship between MMA and mortality after adjustment for sex hormones.

## Data Availability

The datasets of NHANES are available on reasonable request from the website https://www.cdc.gov/nchs/nhanes/index.htm.

## Abbreviations

CKD: Chronic kidney disease
CKD-EPI: Chronic Kidney Disease Epidemiology Collaboration
CVD: Cardiovascular disease
EFT: Empirical free testosterone
FPG: Fasting plasma glucose
GC/MS: Gas chromatography-mass spectrophotometry
HDL-C: High-density lipoprotein cholesterol
HRs: Hazard ratios
ICD-10: International Classification of Diseases,10th Revision
LC-MS/MS: Liquid chromatography-tandem mass spectrophotometry
MMA: Metabolite methylmalonic acid
NHANES: National Health and Nutrition Examination Survey
SHBG: Sex hormone–binding globulin
TC: Total cholesterol
TT: Total testosterone
