# Supplementary material for: Sexual dimorphism in mitochondrial dysfunction and diabetes mellitus: evidence from a population-based cohort study
Source: Diabetol Metab Syndr. 2023 Jun 1;15:114. doi: 10.1186/s13098-023-01090-1 (PMC10234037; doi:10.1186/s13098-023-01090-1)
Supplement: Supplementary file 1 — Figure S1. Standardized levels of Serum Sex Hormones and SHBG by sex and diabetes status. Table S1. HRs for heart-specific mortality per each unit increase in log2-transformed MMA among adults with diabetes. Table S2. Diabetes-related variables at basline by sex. Table S3. The HRs for all-cause and heart-specific mortality per each unit increase in log2-transformed MMA after additional adjustment for diabetes-related variables. Table S4. Correlations of methylmalonic acid with serum sex hormones and SHBG by sex and diabetes status. Table S5. Spearman's rho between methylmalonic acid and sex hormones in females by menopause status. Table S6. Sex-specific HRs for the relationship between MMA and mortality after adjustment for sex hormones. [file 13098_2023_1090_MOESM1_ESM.docx]

## Figure S1. Standardized levels of Serum Sex Hormones and SHBG by sex and diabetes status


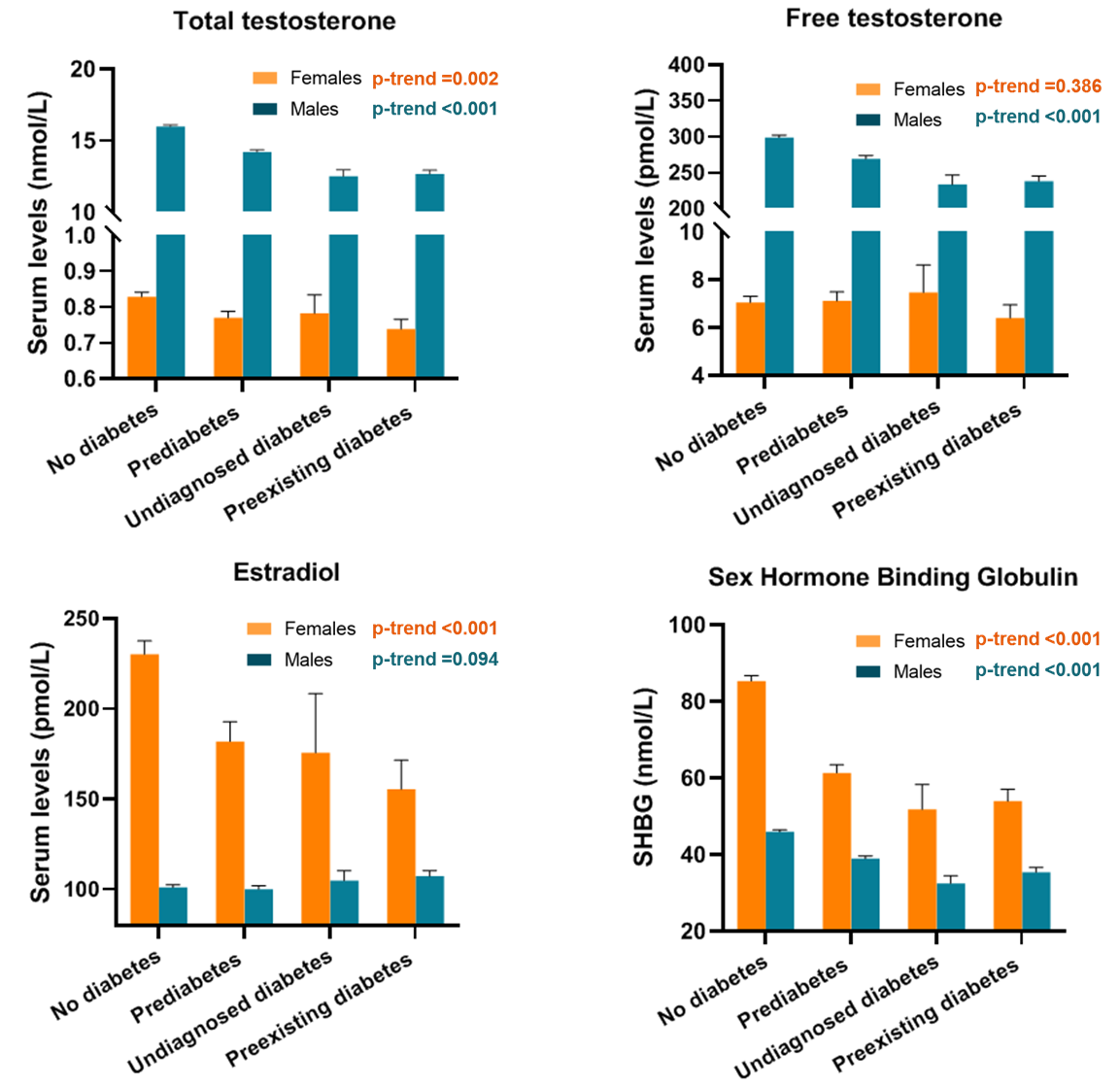


The estimates of sex hormones and SHBG were weighted with the complex sampling design. The difference in biomarkers across diabetic status was assessed by the weighted linear regressi

## Table S1. HRs for heart-specific mortality per each unit increase in log2-transformed MMA among adults with diabetes

|  |  | Events/  person-years^a^ | Mortality rate^b^ | Age-Adjusted |  | Multivariable-Adjusted |  | p-interaction |
| --- | --- | --- | --- | --- | --- | --- | --- | --- |
|  |  |  |  | HR (95%CI) | p value | HR (95%CI) | p value |  |
| No diabetes |  |  |  |  |  |  |  | 0.540 |
|  | Females | 95/73,992 | 0.87 (0.69-1.12) | 1.58 (1.23-2.02) | <0.001 | 1.32 (1.00-1.75) | 0.052 |  |
|  | Males | 132/69,463 | 1.10 (0.89-1.38) | 1.33 (1.00-1.76) | 0.047 | 1.34 (0.99-1.81) | 0.06 |  |
| Prediabetes |  |  |  |  |  |  |  | 0.439 |
|  | Females | 65/18,222 | 2.63 (1.93-3.67) | 1.33 (0.84-2.09) | 0.218 | 1.06 (0.65-1.72) | 0.82 |  |
|  | Males | 94/20,060 | 2.87 (2.26-3.70) | 1.34 (1.06-1.69) | 0.017 | 1.12 (0.82-1.53) | 0.463 |  |
| Undiagnosed diabetes | |  |  |  |  |  |  | 0.220 |
|  | Females | 13/2,518 | 3.97 (2.13-8.25) | 1.71 (1.12-2.62) | 0.014 | 0.80 (0.47-1.38) | 0.418 |  |
|  | Males | 22/3,148 | 6.32 (3.65-11.91) | 2.49 (1.02-6.04) | 0.044 | 1.58 (0.58-4.31) | 0.370 |  |
| Preexisting Diabetes | |  |  |  |  |  |  | 0.045 |
|  | Females | 62/8,720 | 6.99 (5.14-9.75) | 1.38 (1.09-1.75) | 0.009 | 1.23 (0.76-1.97) | 0.391 |  |
|  | Males | 107/8,484 | 11.14 (8.65-14.58) | 1.95 (1.65-2.30) | <0.001 | 1.86 (1.47-2.36) | <0.001 |  |

^a^ unweighted, ^b^ weighted rate per 1000 person-year. Sex-speciﬁc HR (95%CI) was estimated by weighted Cox regression. The age-adjusted model was adjusted for age. The multivariable-adjusted model was additionally adjusted for race/ethnicity, smoking status, physical activity, BMI, hypertension, cancer, CVD, TC/HDL-C ratio, eGFR, HbA1c, and serum cobalamin.

## Table S2. Diabetes-related variables at basline by sex

| **Variables** | **Females** | **Males** | **p value** |
| --- | --- | --- | --- |
| Duration of diabetes (year) | 12.0 ± 0.40 | 11.1 ± 0.40 | 0.118 |
| Diabetic complications (%) | 31.29 | 34.08 | 0.262 |
| Retinopathy (%) | 19.26 | 20.55 | 0.507 |
| Foot ulcer/sore (%) | 3.42 | 4.95 | 0.185 |
| Peripheral neuropathy (%) | 17.29 | 16.72 | 0.763 |
| UACR (mg/g) | 113.6 ± 17.66 | 192.1 ± 21.94 | 0.006 |
| Metformin use (%) | 47.75 | 50.83 | 0.267 |

UACR, urinary albumin-to-creatinine ratio. Categorical and continuous variables were presented as weighted proportions or means (SE) using the chi-square test and Student's t-test, respectively.

## Table S3. The HRs for all-cause and heart-specific mortality per each unit increase in log2-transformed MMA after additional adjustment for diabetes-related variables

|  | All-cause mortality | | | Heart-specific mortality | | |
| --- | --- | --- | --- | --- | --- | --- |
| Sex | HR (95%CI) | p value | p interaction | HR (95%CI) | p value | p interaction |
| Females (n=1250) | 1.08 (0.93-1.26) | 0.319 | 0.031 | 1.12 (0.66-1.89) | 0.669 | 0.036 |
| Males (n=1285) | 1.41 (1.18-1.69) | <0.001 |  | 1.72 (1.33-2.22) | <0.001 |  |

Sex-speciﬁc HR (95%CI) was estimated by weighted Cox regression analyses after adjustment for age race/ethnicity, smoking, physical activity, BMI, hypertension, cancer, CVD, TC/HDL-C ratio, eGFR, HbA1c, Vitamin B12, metformin use, lipid-lowering agents, duration of diabetes, UACR, ACEI/ARBs, and diabetic complications. P for interaction was assessed by the survey-weighted Wald test

## Table S4. Correlations of methylmalonic acid with serum sex hormones and SHBG by sex and diabetes status

|  | **Females** | | | | **Males** | | | |
| --- | --- | --- | --- | --- | --- | --- | --- | --- |
| Sex hormone profiles | No diabetes | Prediabetes | Undiagnosed diabetes | Preexisting diabetes | No diabetes | Prediabetes | Undiagnosed diabetes | Preexisting diabetes |
| Total testosterone, nmol/L  (n= 11,232) | -0.156 | -0.106 | -0.023 | -0.073 | -0.095 | -0.090 | 0.016 | -0.061 |
| Free testosterone, pmol/L  (n= 5,797) | -0.141 | -0.174 | 0.056 | -0.077 | -0.125 | -0.115 | -0.120 | -0.075 |
| Estradiol, pmol/L  (n= 6,309) | -0.229 | -0.353 | -0.230 | -0.185 | -0.062 | -0.089 | 0.061 | 0.111 |
| SHBG, nmol/L  (n= 5,797) | 0.141 | 0.206 | 0.287 | 0.127 | 0.219 | 0.164 | 0.193 | 0.164 |

The study population was restricted to those with at least one eligible indicator including total testosterone, free testosterone, estradiol or SHBG.

SHBG, Sex hormone–binding globulin. Correlation coefficients were estimated by the Spearman method. After Bonferroni correction, the P value ≤ 0.0016 was considered significant.

## Table S5. Spearman's rho between methylmalonic acid and sex hormones in females by menopause status

| Sex hormone profiles | **No menopause** | | **Menopause** | |
| --- | --- | --- | --- | --- |
|  | Samples | Spearman's rho | Samples | Spearman's rho |
| Total testosterone, nmol/L | 2961 | -0.104 | 2197 | -0.056 |
| Free testosterone, pmol/L | 1372 | -0.124 | 1078 | -0.156 |
| Estradiol, pmol/L | 1553 | -0.158 | 1131 | -0.131 |
| SHBG, nmol/L | 1372 | 0.108 | 1078 | 0.139 |

The study population was restricted to those with at least one eligible indicator including total testosterone, free testosterone, estradiol or SHBG.

SHBG, Sex hormone–binding globulin. Correlation coefficients were estimated by the spearman method. All P values ≤ 0.05.

## Table S6. Sex-specific HRs for the relationship between MMA and mortality after adjustment for sex hormones

|  | **Females (n= 2,319)** | | **Males (n= 3,215)** | |
| --- | --- | --- | --- | --- |
|  | HR (95%CI) | p value | HR (95%CI) | p value |
| Basic Model* | 1.38 (0.86-2.21) | 0.182 | 1.30 (1.11-1.53) | 0.001 |
| Plus Total testosterone | 1.36 (0.85-2.19) | 0.200 | 1.31 (1.11-1.54) | 0.001 |
| Plus Free testosterone | 1.36 (0.85-2.18) | 0.204 | 1.31 (1.12-1.54) | 0.001 |
| Plus Estradiol | 1.38 (0.86-2.21) | 0.182 | 1.30 (1.11-1.53) | 0.001 |
| Plus SHBG | 1.36 (0.85-2.20) | 0.202 | 1.30 (1.11-1.53) | 0.001 |
| Plus Free testosterone and Estradiol | 1.36 (0.84-2.17) | 0.207 | 1.31 (1.12-1.54) | 0.001 |

The study population was restricted to those who had completed data on total testosterone, free testosterone, estradiol and SHBG. Sex-speciﬁc HR (95%CI) was estimated by weighted Cox regression analyses for the association of a doubling in MMA with mortality risk. The Basic Model was adjusted for diabetes status, age, race/ethnicity, smoking, BMI, physical activity, menopause (for females), hypertension, cancer, CVD, TC/HDL-C ratio, eGFR, HbA1c, and Vitamin B12.
